# Enthesitis-related arthritis: the clinical characteristics and factors related to MRI remission of sacroiliitis

**DOI:** 10.1186/s12891-022-06028-8

**Published:** 2022-12-03

**Authors:** Jiaoyu Li, Yaju Zhu, Guimei Guo

**Affiliations:** Department of Pediatric Nephrology and Rheumatology, Xinhua Hospital Affiliated to Shanghai Jiao Tong University School of Medicine, Shanghai, 200092 China

**Keywords:** Enthesitis, Juvenile idiopathic arthritis, Sacroiliitis, Remission, Clinical factor

## Abstract

**Background:**

To describe the clinical characteristics and explore the factors related to the MRI remission of sacroiliitis in patients with enthesitis-related arthritis (ERA).

**Methods:**

Patients with ERA from 2018–2022 in our medical center were retrospectively reviewed, which identified according to Pediatric Rheumatology International Trials Organization (PRINTO) criteria. Demographics, clinical characteristics, examinations, and treatments were described. Univariate and multivariate logistic regression models were used to analyze the factors related to MRI remission of sacroiliitis in ERA.

**Results:**

This retrospective study included 160 ERA patients (51.9% male) with a mean onset age of 9.2 ± 3.0 years. There were 144 cases (81.9%) with peripheral arthritis, and the hip, knee, and ankle joints were the most commonly involved joints. Enthesitis occurred in 48 cases (30.0%), and sacroiliitis occurred in 142 cases (88.5%) at diagnosis. Human leukocyte antigen (HLA)-B27 was positive in 33 cases (17.1%), and acute uveitis occurred in 3 cases (1.9%). The majority of patients (93.7%) were treated with disease-modifying anti-rheumatic drugs (DMARDs), and 60% with biologics. Among 62 patients with MRI-defined sacroiliitis, 27 (43.5%) cases showed improvement in the sacroiliac joint lesion after treatment. Multivariate logistic regression analysis showed that duration from onset to diagnosis of less than 3 months (OR = 3.609, 95% CI: 1.068–12.192) and active joints of more than 4 (OR = 4.916, 95% CI: 1.006–24.037) were independent factors.

**Conclusion:**

We highlighted differences in ERA clinical characteristics. Patients with a shorter diagnosis time and more joint involvement improved more significantly in sacroiliac joint lesions after treatment.

## Introduction

Juvenile idiopathic arthritis (JIA) is the most common chronic rheumatic disease in children [1]. According to the Pediatric Rheumatology International Trials Organization (PRINTO) classification of 2018, JIA can be divided into six categories based on clinical features [2]. Enthesitis-related arthritis (ERA) is a subtype of JIA, characterized as the involvement of peripheral joints, entheses, and the axial skeleton, and is considered the counterpart of adult spondyloarthropathies [3]. The etiology of ERA remains unclear, although evidence shows that the interactions of genetic elements and environmental factors might cause the disease [3]. HLA-B27 is the most commonly mentioned genetic factor involved in the pathogenesis of ERA. HLA-B27 is associated with antigen processing and presentation [4]. Infections, gut microbiomes, and inflammation of the bowel wall are also suspected in the etiology of ERA [5]. A detailed history, careful physical examination, and imaging modalities are important for the diagnosis of ERA. Nonsteroidal anti-inflammatory drugs (NSAIDs), disease-modifying antirheumatic drugs (DMARDs), and biological agents are recommended for the treatment of ERA [6].

Research in Europe, the Middle East, and North America shows that oligoarthritis is the most common category (30–50%) in JIA patients, and ERA accounts for only 5–15% [7, 8]. However, some studies have demonstrated that the prevalence in Asians is significantly higher, up to about 30–40% [9, 10], which might be due to differences in ethnic groups [11]. Patients with ERA tend to have higher pain intensity, more chronic disease, and poorer health status compared to their counterparts with other categories of JIA [9, 12, 13]. Due to the relatively low prevalence, only a few studies have focused on detailed ERA clinical characteristics, treatments, and outcomes. ERA patients with axial joint involvement are generally asymptomatic, and the manifestation (inflammatory back pain and/or sacroiliac joint tenderness) occurs later in disease, compared with adult patients [14]. About 30–50% of patients with ERA are diagnosed with clinical or radiologic sacroiliitis [15, 16], and are more likely to have a poorer treatment response [9]. Active inflammatory lesions of the sacroiliac joints could be relieved after treatment, and remission based on clinical symptoms and MRI examination are not completely concordant [17].

There is still a lack of relevant epidemiological data on the incidence of ERA in China. Thus, the purpose of this study was to summarize the clinical characteristics, treatment, and outcome of ERA. Further, the remission of sacroiliitis in some ERA patients is often confirmed by MRI after treatment. Thus, we also sought to explore the factors related to the MRI-confirmed remission of sacroiliitis. The findings from this study may provide insights into improving the prognosis and quality of life of children through early diagnosis and intervention.

## Study design and participants

### Study population

In this retrospective study, 264 patients diagnosed with JIA according to the 2018 PRINTO classification criteria were recruited from Xinhua Hospital (affiliated with the Shanghai Jiao Tong University School of Medicine) from December 2018 to May 2022. Of the sample, 160 patients fulfilled the classification criteria for ERA. Males accounted for 51.9%, and the mean onset age was 9.2 ± 3.0 years. For all patients, demographic details, family history, clinical manifestation, laboratory and radiographic examinations, and medications were routinely recorded. The study protocol and ethical approval were obtained from the Ethics Committee of Xinhua Hospital affiliated with the Shanghai Jiao Tong University School of Medicine (XHEC-D-2022–212). All patients and their parents signed informed consent forms.

### Definitions

The classification of enthesitis-related JIA stipulates: (1) peripheral arthritis and enthesitis; (2) arthritis or enthesitis, plus ≥ 3 months of inflammatory back pain and sacroiliitis on imaging; (3) arthritis or enthesitis and two of the following: (a) sacroiliac joint tenderness; (b) inflammatory back pain; (c) presence of HLA-B27 antigen; (4) acute (symptomatic) anterior uveitis; and (5) history of a SpA in a first-degree relative [2]. If peripheral arthritis was present, it should have persisted for at least 6 weeks to be classified as ERA. The duration from onset to diagnosis was defined as the date the patient developed clinical arthritis until a definite diagnosis was made. Peripheral arthritis was defined as swollen joints or joints with a limited range of motion accompanied by pain on motion and/or tenderness [18]. The active joint count (AJC) was defined as the number of joints with peripheral arthritis. Enthesitis was defined as inflammation localized to a tendon, ligament, or joint capsule insertion site to bone, with the clinical symptoms of tenderness and swelling at specific sites, which could be confirmed by MRI [19–21]. Clinical evidence of sacroiliitis was defined as patients suffering from low back pain and stiffness for more than 3 months, limitation of motion of the lumbar spine, or chest expansion [22]. MRI-defined sacroiliitis was defined by the presence of inflammation (bone marrow edema, joint space enhancement, or erosions/sclerosis) in the sacroiliac joint visualized by MRI [23], with or without the manifestation of clinical sacroiliitis. The MRI remission of sacroiliitis was defined as the lesion of the sacroiliac joint relieved compared with the previous image after treatment for over 6 months. The Juvenile Spondyloarthritis Disease Activity (JSpADA) is a reliable score tool for measuring disease activity in ERA [24], and it consists of eight items (morning stiffness, patient reported pain, active joints count, active enthesitis count, clinical sacroiliitis, abnormal back mobility, uveitis, and inflammatory biomarkers). JSpADA scores range from 0 to 8, with higher scores indicative of more active disease [25].

### Statistical analysis

Statistical analysis was performed using SPSS (version 22.0; IBM, New York, NY) and GraphPad Prism (version 8.1.1). Data are presented as median with mean ± SD for continuous variables, and n (%) for categorical variables. Independent sample t-tests and chi-square tests were performed where appropriate. A multivariate logistic regression model was used to examine the independent predictors of MRI remission of sacroiliitis, which included variables with a *P* < 0.1 in the univariate analysis [26]. Results were considered statistically significant at *P* < 0.05.

## Results

**Fig. 1:**
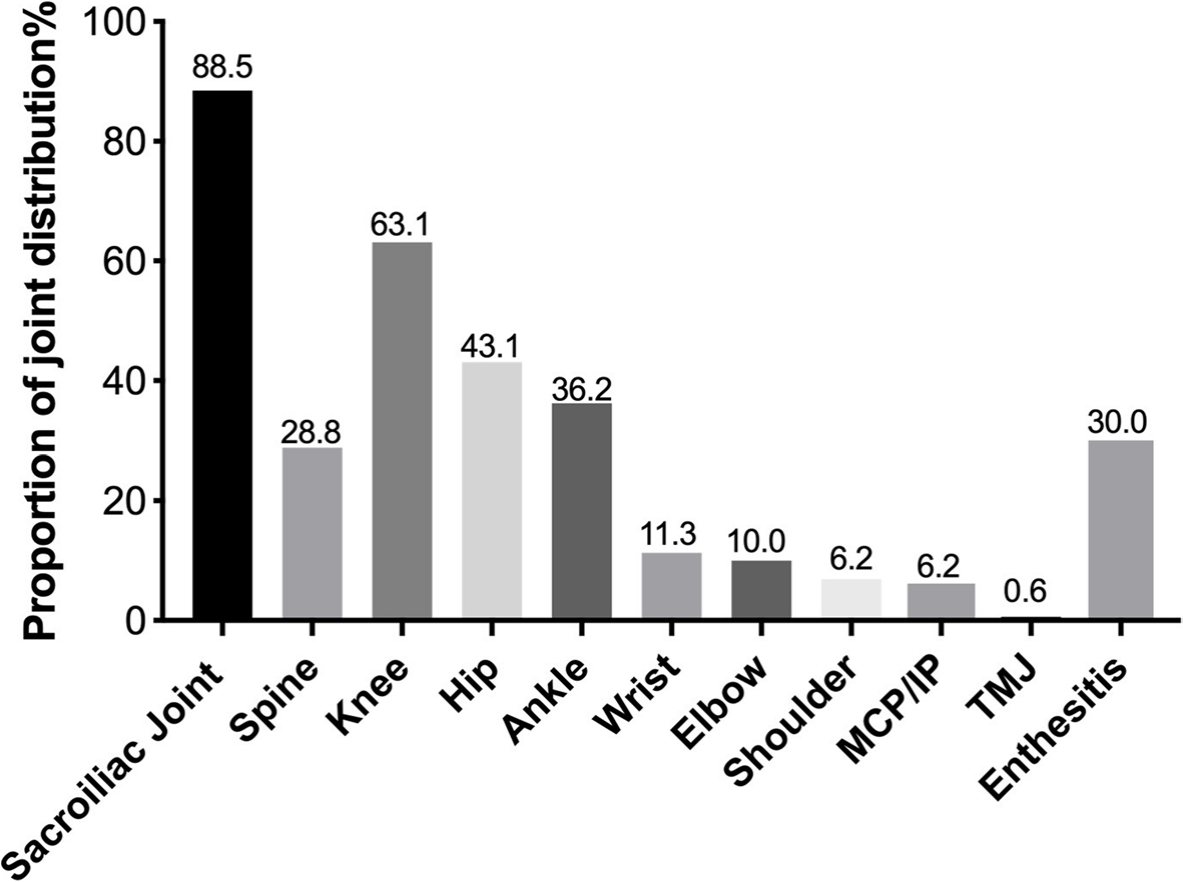
Proportion of affected joint distribution (%) at diagnosis. MCP, metacarpophalangeal joint; IP, interphalangeal joint; TMJ, temporomandibular joint

From December 2018 to May 2022, in our study, 264 patients were diagnosed with JIA, of which 160 (60.6%) fulfilled the 2018 PRINTO classification criteria for ERA. The demographic and clinical characteristics are shown in Table 1. There was no difference between males (51.9%) and females, and the patients had a mean onset age of 9.2 ± 3.0 years. The mean diagnosis time was 7.6 ± 12.4 months. A total of 14 patients had a family history, and 25 patients had trigger factors before the clinical feature onset, including trauma in joints and respiratory or digestive infections. Among the patients with ERA, 90% had peripheral arthritis at diagnosis, and the median number of active joints at diagnosis was 2 (IQR 1–4). The knee (63.1%), hip (43.1%), and ankle (36.2%) were among the common joints involved (Fig. 1). Enthesitis developed in 48 patients (30%), and Achilles tendon enthesis remained the most commonly involved. There were 142 patients (88.5%) with defined sacroiliitis according to MRI, but only 36 patients presented with low back pain at diagnosis. Only 1.9% of the patients developed anterior uveitis. In total, 15 patients with digestive symptoms were confirmed to have no inflammatory bowel disease by gastrointestinal endoscopy. Of note, only 20% of patients were HLA-B27 positive. The mean ESR at diagnosis was 22.4 ± 26.9 mm/h and CRP was 11.1 ± 26.9 mg/L. The mean JSpADA score was 2.3 ± 0.73. A quarter of the patients received NSAIDs, and almost all ERA patients received DMARDs (97.3%). The majority of the patients (68.1%) had methotrexate (MTX). Moreover, 7.5% of the patients required corticosteroids, and only one patient received intra-articular steroid injections. Anti-TNF (aTNF) was the most commonly used biologic (60%).

**Table 1:** The clinical characteristics and outcome of ERA

**Characteristics**	(*N* = 160)
**Demographic characteristics**	
Male sex, n (%)	83 (51.9)
Age at onset, y	9.2 ± 3.0
Duration from onset to diagnosis, m	7.6 ± 12.4
Duration at follow-up visit, m	18.4 ± 11.9
**Disease characteristics**	
Peripheral arthritis at diagnosis, n (%)	144 (90.0)
AJC, n (IQR)	2 (1, 4)
Enthesitis, n (%)	48 (30.0)
Back Pain, n (%)	36 (22.5)
Family history, n (%)	14 (8.6)
A history of trauma, n (%)	17 (10.6)
Infection, n (%)	8 (5.0)
Uveitis, n (%)	3 (1.9)
Inflammatory bowel disease, n (%)	0 (0)
WBC, 10^9/L	6.8 ± 2.0
ESR at diagnosis, mm/h	22.4 ± 26.9
CRP at diagnosis, mg/L	11.1 ± 26.9
TNF-a, pg/ml	25.0 ± 49.7
HLA-B27 (+), n (%)	33 (20.6)
ANA (+), n (%)	23 (14.4)
RF (+), n (%)	1 (0.6)
FER, ug/L	71.7 ± 73.3
IgG, g/L	12.0 ± 3.3
IgA, g/L	1.7 ± 0.9
IgM, g/L	1.3 ± 0.6
IgE, g/L	180.8 ± 343.9
JSpADA	2.3 ± 0.73
**Mediation**	
NSAIDs, n (%)	43 (26.8)
Steroids, n (%)	12 (7.5)
Intraarticular steroids injection, n (%)	1 (0.6)
Methotrexate, n (%)	109 (68.1)
Sulfasalazine, n (%)	48 (30.0)
Leflunomide, n (%)	11 (6.9)
Biologics, n (%)	96 (60.0)
DMARDs + Biologics, n (%)	91 (56.9)

*AJC* active joint counts, *WBC* white blood cell, *ESR* Erythrocyte Sedimentation Rate, *CRP* C-Reactive Protein, *GPI* glucose phosphate isomerase, *ANA* antinuclear antibodies, *RF* rheumatoid factors, *FER* Ferritin, *JSpADA* Juvenile Spondyloarthritis Disease Activity, *NSAIDs* Non-Steroidal Antiinflammatory Drugs, *DMARDs* disease-modifying antirheumatic drugs, *m* month, *y* year

**Fig. 2:**
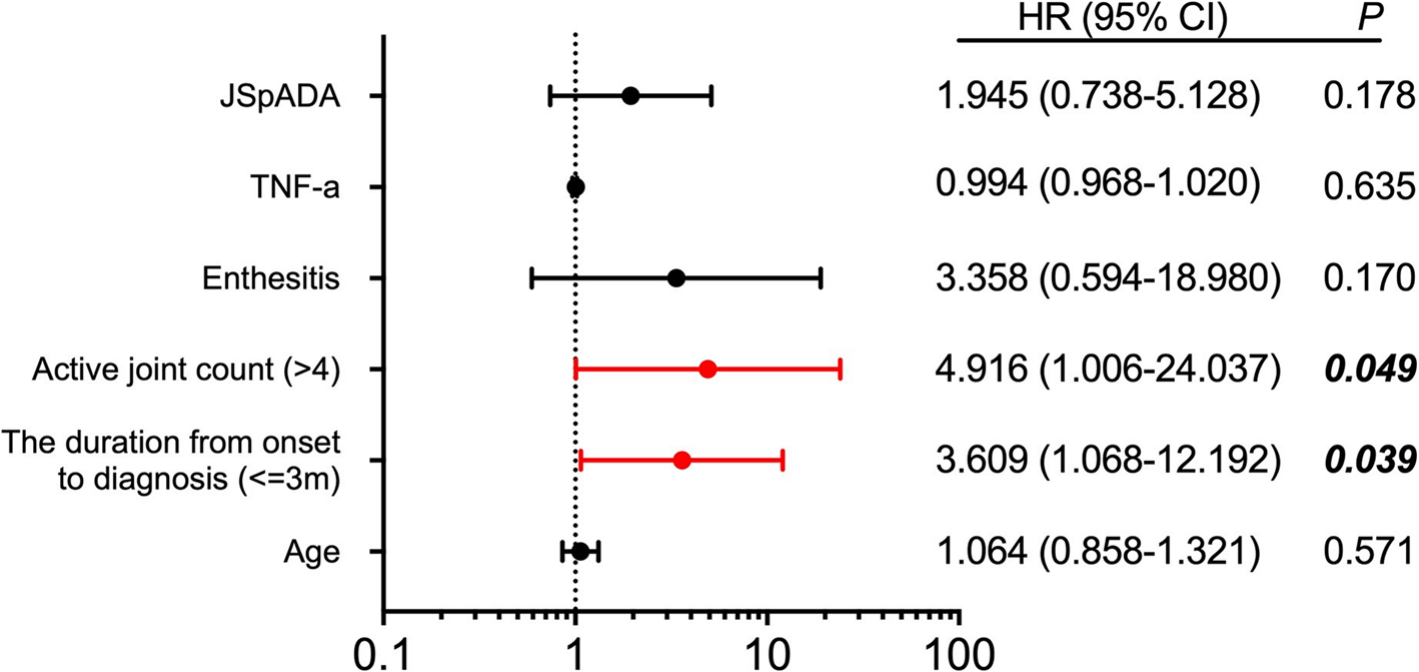
Multivariate logistic regression analysis of MRI remission of sacroiliitis. Multivariable risk factor and 95% confidence interval for MRI remission. AJC (> 4) (OR = 4.916) and diagnosis time (≤ 3 m) (OR = 3.609) were independent risk factors (*P* < 0.05)

Among 62 patients with sacroiliitis who underwent MRI after treatment for over 6 months, 27 patients had improved sacroiliac joint lesions. The results of the univariate analysis for related factors are shown in Table 2. The diagnosis time of ≤ 3 months, AJC > 4, with enthesitis, high TNF-a levels and JSpADA scores were potential predictors of ERA (*P* < 0.1). The multivariable analysis is shown in Fig. 2. The duration from onset to diagnosis of ≤ 3 months (OR = 3.609; 95% CI: 1.068–12.192) and AJC > 4 (OR = 4.916; 95% CI: 1.006–24.037) significantly correlated with the improvement of sacroiliitis in MRI.

**Table 2:** The univariate analysis analysis for related factors of MRI remission

	Non-remission (*N* = 35)	Remission (*N* = 27)	*P* value
Male sex, n (%)	19 (54.3)	16 (59.3)	0.304
Age at onset, y	9.3 ± 2.7	9.4 ± 3.6	0.936
Duration from onset to diagnosis ≤ 3 m, n (%)	13 (37.1)	19 (70.4)	0.009
Family history, n (%)	1 (2.9)	3 (11.1)	0.215
AJC > 4, n (%)	4 (11.4)	9 (33.3)	0.037
Back pain, n (%)	7 (20)	6 (22.2)	0.537
Hip arthritis, n (%)	19 (54.3)	13 (48)	0.412
Knee arthritis, n (%)	15 (42.9)	16 (59.3)	0.153
Enthesitis, n (%)	13 (37.1)	4 (14.8)	0.046
ESR at diagnosis, mm/h	20.5 ± 26.9	23.8 ± 29.6	0.645
CRP at diagnosis, mg/L	15.6 ± 42.0	11.7 ± 26.1	0.673
TNF-a, pg/ml	30.9 ± 41.0	17.2 ± 21.6	0.095
FER, ug/L	89.6 ± 105.9	100.1 ± 122.4	0.772
IgG, g/L	11.5 ± 3.1	12.8 ± 4.1	0.201
IgA, g/L	1.6 ± 0.7	1.8 ± 1.0	0.394
IgM, g/L	1.3 ± 0.9	1.3 ± 0.5	0.960
IgE, g/L	208.9 ± 295.6	224.5 ± 526.0	0.922
B27 (+), n (%)	8 (22.9)	6 (22.2)	0.600
ANA (+), n (%)	2 (5.7)	5 (18.5)	0.121
JSpADA	2.2 ± 0.6	2.5 ± 0.8	0.058
Biogics, n (%)	27 (77.1)	22 (81.5)	0.463

*AJC* active joint counts, *WBC* white blood cell, *ESR* Erythrocyte Sedimentation Rate, *CRP* C-Reactive Protein, *GPI* glucose phosphate isomerase, *ANA* antinuclear antibodies, *RF* rheumatoid factors, *FER* Ferritin, *JSpADA* Juvenile Spondyloarthritis Disease Activity, *NSAIDs* Non-Steroidal Antiinflammatory Drugs, *DMARDs* disease-modifying antirheumatic drugs, *m* month, *y* year

## Discussion

In Western countries, oligoarthritis has proven to be the most common type of JIA, whereas ERA is the most common type in non-Western populations. In our cohort, we found that ERA had a significantly higher prevalence of up to 60%. According to a multiethnic cohort, ethnicity is a risk factor in the epidemiology of JIA, and patients of Asian origin account for the most predominant group [11]. A retrospective multicenter study conducted in the United States described the clinical characteristics of ERA patients as predominantly male (64%), with a mean age of 11 years at diagnosis, approximately 60% HLA-B27 positivity, and a pauciarticular onset [27]. Our results are not fully consistent with these findings due to differences in classification criteria. No male preponderance was observed in our cohort, which was different from those of previous studies that reported a male proportion of 63–91% [9, 10, 16, 28, 29]. Further, in our study, HLA-B27 positivity was relatively lower than previous reports of 43–90% [9, 10, 16, 28, 29]. HLA-B27 positivity has been associated more with males and ethnic variation [27]. It is also related to a higher active joint count, more sacroiliitis, and higher disease activity [30]. Peripheral arthritis was present in 90% of our patients, similar to other cohorts [9, 27, 29]. Enthesitis was observed in only 30%, which was similar to that of a Singapore cohort but less than that of other cohorts (50–75%) [9, 10, 27]. The knee was the most commonly affected joint, consistent with previous studies (37–52%) [3]. However, data from Singapore showed that the hip was the most common joint involved, up to 60% [15]. NSAIDs and DMARDs were first-line medications for the treatment recommendation for ERA in our study, which is similar to previous studies [3]. Methotrexate (MTX, 68.1%) was the most common DMARDs. The use of biologic agents has increased in the treatment of children with sacroiliitis [31], but it might also increase the risk of cancer development [32].

Compared to other categories of JIA, patients with ERA always experienced a delay in diagnosis and suffered worse functional impairments. Inflammatory back pain and imaging criterion of sacroiliitis are emphasized in the PRINTO classification. Signs of sacroiliitis are closely related to poorer outcomes in ERA patients [9]. According to recent research, hip arthritis, HLA-B27 positivity, older age at onset, female gender, the number of active joints, and enthesitis increased the risk of sacroiliitis [10, 15, 33]. With the application of the MRI detection method, changes in the axial joint can be observed at an early stage [34, 35]. In our ERA cohort, sacroiliitis was defined by MRI in 142 patients (88.7%) at diagnosis, and 9 patients developed sacroiliitis during the follow-up period, but only 36 patients had inflammatory spinal pain during the disease. As sacroiliitis in ERA tends to be asymptomatic, careful physical examination and MRI are needed. The prevalence of sacroiliitis was much higher in our study than in other reports (16–55%) [9, 10, 27, 36]. In the cohorts of previous studies, only patients with clinical sacroiliitis and (or) HLA-B27-positive status would undergo MRI of the sacroiliac joint; however, we routinely obtained MRI in our JIA patients to avoid a delayed detection of sacroiliac joint lesions, which might contribute to the discrepancy.

About two-thirds of our patients with ERA had non-active disease at their last visit, with a follow-up duration of 5 years [37]. Among patients with MRI-defined sacroiliitis, MRI-confirmed remission in the respective location was achieved by 65% of patients one year after biologics treatment [17]. Our study revealed that approximately 40% of the patients had improved of sacroiliac joint lesions, as confirmed by MRI. Patients with a time from onset to diagnosis of less than 3 months tended to achieve MRI remission, which brought early diagnosis and aggressive treatment during the window of opportunity to improve long-term disease outcomes [38]. MRI remission was also achieved in patients with more active joint counts (> 4). Extensive peripheral joint symptoms might cause more concern for parents and children, and they tend to seek medical attention earlier. Further, some data showed that there were no differences between the number of active joints among active and non-active patients [9].

However, our study also had several limitations. First, this is a retrospective, single-center observational study, which might contribute to some selection bias. Second, the follow-up time was not long enough, which led to a loss of follow-up clinical information. Third, the MRI remission of sacroiliitis lacked quantitative assessment tools to assess the changes accurately.

This is the largest study in China that attempted to characterize the clinical characteristics of ERA and explored the factors associated with the improvement of sacroiliitis. Our findings indicate that ERA was the most common JIA category in China. Compared with previously published cohorts, some differences in disease features were observed: no prominent differences between males and females, a lower prevalence of HLA-B27, and more sacroiliac joint involvement. Patients with more joint involvement and a shorter diagnosis time tended to achieve improvement in sacroiliitis, as confirmed by MRI.

## Data Availability

The datasets generated and/or analyzed during the current study are not publicly available due to privacy and ethical issues, but are available from the corresponding author on reasonable request.

## Abbreviations

ERA: Enthesitis-related arthritis
PRINTO: Pediatric Rheumatology International Trials Organization
HLA: Human Leukocyte Antigen
DMARDs: Disease modifying anti-rheumatic drug
JIA: Juvenile idiopathic arthritis
AJC: Active joint count
JSpADA: Juvenile spondyloarthritis disease activity
MTX: Methotrexate
aTNF: Anti-TNF
